# Acetone–Gasoline Blend as an Alternative Fuel in SI Engines: A Novel Comparison of Performance, Emission, and Lube Oil Degradation

**DOI:** 10.1021/acsomega.2c08271

**Published:** 2023-03-13

**Authors:** Muhammad Usman, Talha Khan, Fahid Riaz, Muhammad Ali Ijaz Malik, Muhammad Tahir Amjad, Muhammad Haris Shah, Waqar Muhammad Ashraf, Jaroslaw Krzywanski, Wojciech Nowak

**Affiliations:** † Mechanical Engineering Department, University of Engineering and Technology, G.T. Road, 54890 Lahore, Pakistan; ‡ Mechanical Engineering Department, Abu Dhabi University, P.O. Box 59911 Abu Dhabi, United Arab Emirates; § Sargent Centre for Process Systems Engineering, Department of Chemical Engineering, University College London, Torrington Place, WC1E 7JE London, U.K.; ∥ Faculty of Science and Technology, Jan Dlugosz University in Czestochowa, 42-217 Czestochowa, Poland; ⊥ Faculty of Energy and Fuels, AGH University of Science and Technology, Mickiewicza 30, 30-059 Krakow, Poland

## Abstract

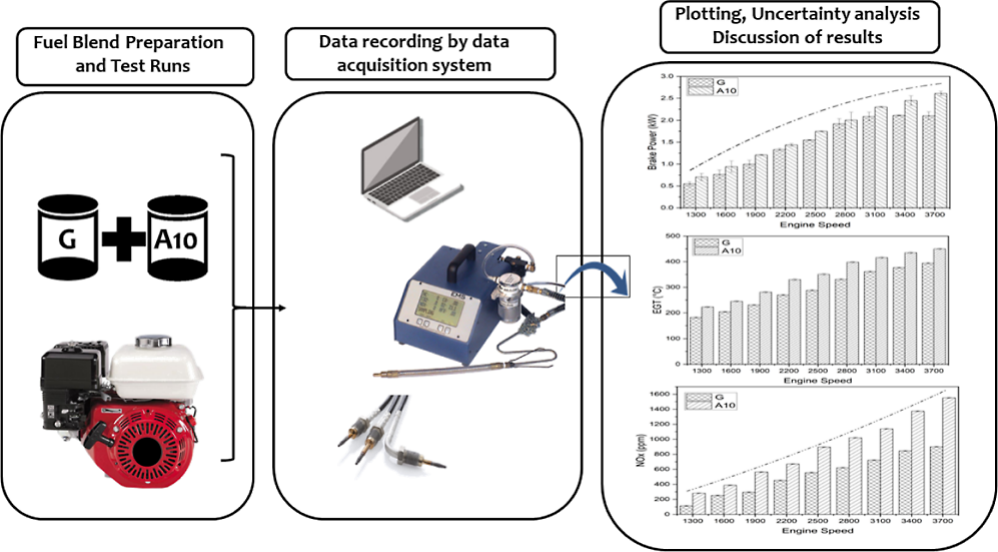

The disproportionate use of petroleum products and stringent exhaust emissions has emphasized the need for alternative green fuels. Although several studies have been conducted to ascertain the performance of acetone–gasoline blends in spark-ignition (SI) engines, limited work has been done to determine the influence of fuel on lubricant oil deterioration. The current study fills the gap through lubricant oil testing by running the engine for 120 h on pure gasoline (G) and gasoline with 10% by volume acetone (A10). Compared to gasoline, A10 produced better results in 11.74 and 12.05% higher brake power (BP) and brake thermal efficiency (BTE), respectively, at a 6.72% lower brake-specific fuel consumption (BSFC). The blended fuel A10 produced 56.54, 33.67, and 50% lower CO, CO_2_, and HC emissions. However, gasoline remained competitive due to lower oil deterioration than A10. The flash-point and kinematic viscosity, compared to fresh oil, decreased by 19.63 and 27.43% for G and 15.73 and 20.57% for A10, respectively. Similarly, G and A10 showed a decrease in total base number (TBN) by 17.98 and 31.46%, respectively. However, A10 is more detrimental to lubricating oil due to a 12, 5, 15, and 30% increase in metallic particles like aluminum, chromium, copper, and iron, respectively, compared to fresh oil. Performance additives like calcium and phosphorous in lubricant oil for A10 decreased by 10.04 and 4.04% in comparison to gasoline, respectively. The concentration of zinc was found to be 18.78% higher in A10 when compared with gasoline. A higher proportion of water molecules and metal particles were found in lubricant oil for A10.

## 1 Introduction

The energy imbalance instigated by the excessive use of nonrenewable fuels in the automotive industry in specific and the industrial sector in general is an alarming issue.^1−5^ Moreover, the shambolic state of global warming and pollution associated with exhaust emissions and engine lubricating oil disposal is equally unignorable.^6,7^ Among all fuels, hydrocarbon fuel is majorly responsible for environmental pollution.^8^ 18% of global primary energy is utilized by the transport sector and is primarily accountable for 23% of global CO_2_ emissions, eventually leading to consequences of global warming.^9^ Many research studies have been rendered to assess the remaining lifecycle of fossil fuels, and shocking results unveiled their remaining estimated life to be the next 40 years.^10,11^ Consequently, researchers have long been firmly looking for alternative renewable energy resources that are performance-efficient and friendly to the environment.^12,13^ Fuels might be promising in terms of exhaust emissions and engine performance. However, the deterioration imparted to the engine lubricating oil could be enormous and needs to be adequately accounted for.^14,15^

The use of alternative fuels has been getting exceptionally common for both spark ignition (SI) and compression ignition engines over the past decades.^16^ Usman et al. comparatively evaluated the effect of liquefied petroleum gas (LPG), gasoline, and LPG-hydroxy gas (HHO) blends on SI engines and deduced that a hybrid mixture of LPG-HHO showed reduced emissions and improved performance compared to neat LPG.^17^ Similarly, Ahmed et al. considered the performance and emissions with methanol addition to gasoline in blend percentages of 3, 6, 9, 12, 15, and 18%.^18^ They inferred that among all tested blends, the best performance and least emissions were found for M12 (12% by volume methanol in gasoline). Among the many alternative blended fuels that are being researched, acetone is the prominent name. It could be used as a blended fuel and can outperform pure gasoline in terms of performance and emissions.^19^ It has oxygen content, low knock tendency, and high flame speeds.^18,20^ In this context, Elfasakhany^21^ investigated the influence of acetone addition on gasoline in the range of 3–10% for exhaust emission and efficiency. He concluded that all the blended fuels showed improved performance and reduced emissions. However, 10% acetone by volume addition to pure gasoline (A10) showed the most promising results. The torque, exhaust gas temperature (EGT), brake power (BP), volumetric efficiency (VE), and cylinder pressure increased by 2.1, 5, 5.2, 3.2, and 10.5% for A10, respectively, while the torque, EGT, BP, VE, and cylinder pressure increased by 0.45, 0.8, 1.3, 0.9, and 2.3% for A3 (3% by volume acetone blended in 97% by volume gasoline). The CO, CO_2_, and UHC decreased by 40, 29.5, and 35% for A3, respectively. The CO, CO_2_, and UHC declined by 46.7, 35.5 and 31.8% for A10, respectively.

Alahmer^22^ experimented by employing two acetone–gasoline fuel blends (A5 and A10). He found the most optimal results for A10 in terms of higher VE, BP, BSFC, and BTE by 7.2, 4.39, 5.2, and 6.9%. However NO_*x*_, CO, UHC, and CO_2_ emissions were reduced by 6.6, 26.3, 30.3, and 4.4%, respectively. Similarly, in another study, acetone–gasoline, isobutanol, and methanol were comparatively evaluated with neat gasoline for performance and emissions.^23^ The results identified that the acetone–gasoline blend was the least detrimental in terms of CO and hydrocarbon emissions. In addition, to binary blends of acetone, the effect of a ternary blend—water comprising acetone–butanol–ethanol gasoline in an SI engine—was studied by Li et al. The acetone–butanol–ethanol in 29% water content (ABE29W) blend showed a 3.1—8.2% higher torque compared to pure gasoline.^24^ Veza et al.^25^ piloted a review study in order to compare the performance of acetone and its blend with butanol–ethanol–gasoline. They found the highest octane rating in acetone as compared to butanol, ethanol, and gasoline. The octane rating is eventually responsible for antiknock characteristics and allows the engine to operate at higher compression ratios in order to operate more efficiently. Alahmer^26^ found suitable outcomes for A10 with a 4.39% improvement in BP along with a 6.6, 26.3, and 30.3% decline in NOx, CO, and UHC emissions. Kantaroğlu et al.^27^ compared the physicochemical attributes of acetone and gasoline in order to conclude the better combustion behavior of fuel inside the engine. They found a higher laminar flame speed of acetone (42.5 cm/s) in comparison with gasoline (33.0 cm/s) which improves combustion efficiency through rapid flame propagation. Acetone possesses higher heat of vaporization which results in cool air during intake, resulting in higher air density and VE. Wu et al.^28^ used acetone as a cosolvent in order to improve the phase stability of butanol, ethanol, and gasoline. They used ABE30 (10% acetone, 10% butanol, and 10% ethanol in 70% gasoline) as fuel in the SI engine. They found 1.4% higher BTE at the cost of 14% lower CO, 9.7% lower HC, and 23.4% lower NO_*x*_ emission.

Malik et al. examined the effect of a methanol gasoline blend on the altered physicochemical properties of engine lubricating oil. The conclusions revealed that the oxygenated blended fuel exhibited a higher decline of 18.78% in kinematic viscosity than neat gasoline (11.61%).^29^ Similarly, Usman and Hayat made a comparative assessment of compressed natural gas (CNG) and high-octane gasoline’s effect on lubricating oil deterioration and considered the property variations, wear debris concentration, and additive depletion. The results vouched for CNG as less damaging to engine oil owing to 3.2, 4.9, and 9.5% less reduction in total base number (TBN), viscosity, and flash point, respectively, compared to neat gasoline. Similarly, the additive depletion rate of Fe, Cu, Cr, and Zn was higher for gasoline than for CNG.^30^ Moreover, in a similar study, two different grades of gasoline with octane numbers 97 and 92 were relatively evaluated for lube oil degradation. The comprehensive analysis of properties and wear debris rendered higher-octane-number fuel unfavorable for lubricating oil.^31^

Many successful research investigations have been made to optimize engine performance through alternative fuels. However, a meticulous effort is needed to examine the influence of an acetone–gasoline blend on lubricant oil under a safe limit. Fuel consumption depends on several factors like lubricant oil chemistry (additives and viscosity grades), engine operating points, and the temperature of the lubricant oil.^32^ Moreover, the viscosity of the lubricant oil plays an important role in the effective performance of lubricant oil and, ultimately, in fuel consumption.^33^ The oxidation or contamination in deteriorated lubricant oil results in an increment in viscosity, as studied in ref (34). Moreover, a lubricant oil with extremely lower viscosity loses oxidation stability at elevated temperatures and its molecules break down early, as discussed in ref (35). The physicochemical attributes of lubricant oils greatly vary with the combustion chemistry. The oxidation rate is directly proportional to temperature, As the lubricant starts degrading when the temperature exceeds 60 °C.

Furthermore, contaminants (moisture and metallic particles) expedite oxidation. The moisture in the lubricant oil is mainly responsible for corrosion, and polymerization and cracking may occur upon thermal degradation of the lubrication oil.^30^ The leakage fuel in the crankcase causes a reduction in the viscosity of the lubricant oil. The decline in viscosity may lead to weaker films and metallic contact, which ultimately result in wear and tear rate owing to failure in sustaining higher loads.^36^

The cited literature reveals that acetone has long been considered a potential alternative blended fuel in SI engines. However, nothing so far has been reported regarding the influence of fuel on engine lubricating oil deterioration. The previous studies indicate that acetone has been used as a cosolvent when butanol and ethanol are blended in the gasoline in order to ensure phase stability. The gap was identified to investigate the performance of acetone thoroughly in the engine including performance, emissions, and lubricant oil deterioration. Although some scientists reported the best-optimized results for the A10 blend, its impact on lubricant oil still needs to be determined. In this work, for the first time, two liquid fuels—pure gasoline and 10% by volume addition to the gasoline (A10) are evaluated for performance, emission, and lubricating oil deterioration in a SI engine. The oil damage for both cases was assessed through variations in physical and chemical attributes like viscosity, flash point (FP), TBN, and water content. The presence of foreign metallic particles like aluminum, chromium, copper, and iron, and the depletion rate of performance additives like calcium, phosphorus, and zinc also served as key factors in assessing the performance of lubricant oil when run on gasoline and A10 subsequently. Therefore, the variation of the above-mentioned factors can serve as the base to examine the impact of both fuels (gasoline and A10) on lubricant oil. A wide-ranging assessment of oxygenated fuel was carried out to check all possibilities of mentioned fuel as a viable solution to combat depleting fossil fuels and ever-growing emissions. Thus the presented cutting-edge research has an impact not only on specific research questions but also on global issues.

## 2 Materials and Methods

The engine used for the experimentation was a 163 cc, air-cooled, commercially available SI engine. The attributes of the engine are listed in Table 1.

**Table 1:** Test Engine Specifications

specification	description
number of strokes	04
net power (kW)	3.6
tank (L)	3.1
maximum torque (Nm)	10.3
cooling mode	air-cooled
bore (mm)	68
compression ratio	8.5:1

**Figure 1:**
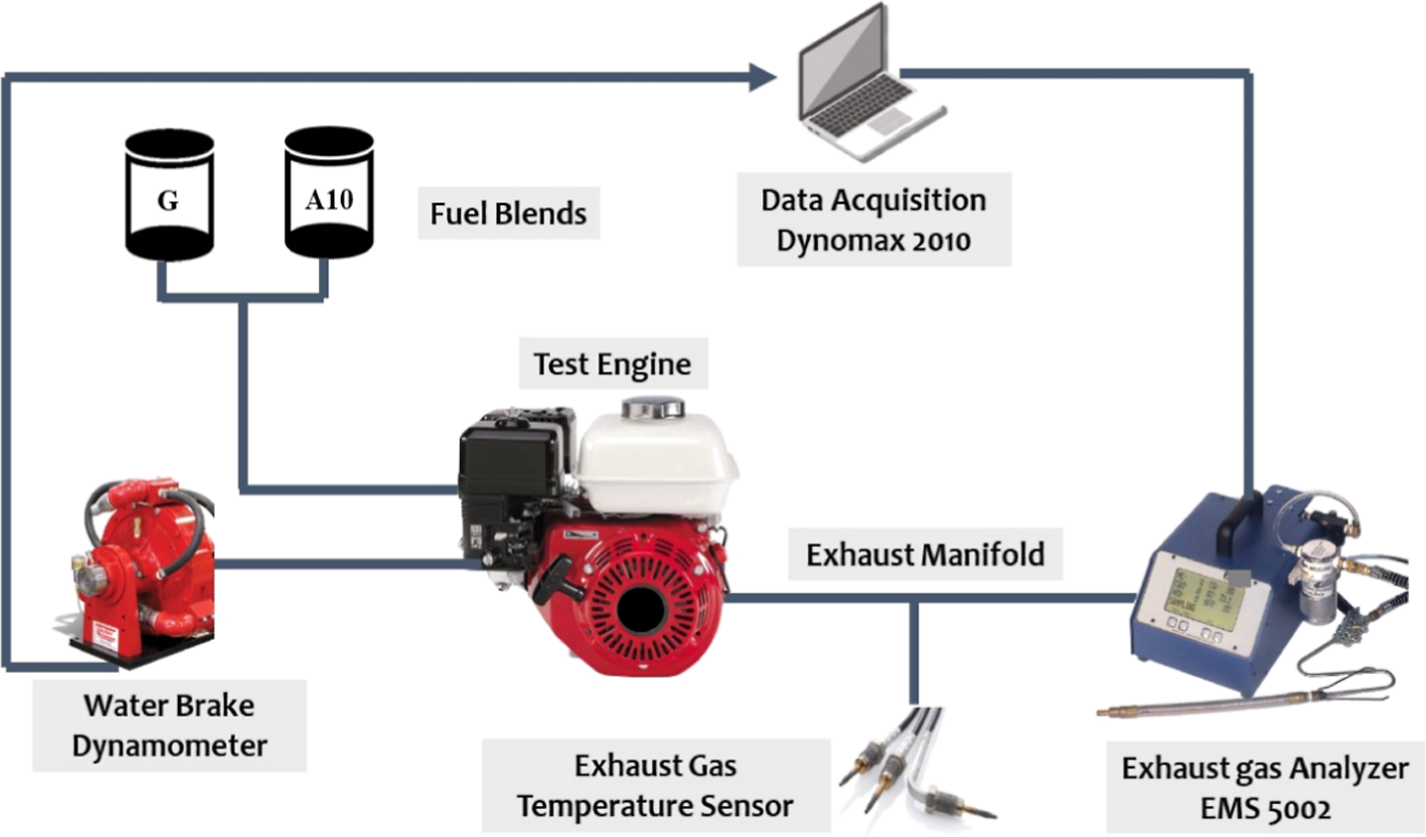
Schematic of the experimental setup.

A comprehensive representation of the experimental setup, which comprised a dynamometer (DYNOMAX), measuring cylinder, EGT sensor, and environmental pollutant analyzer, is shown in Figure 1. A dynamometer is attached to the engine via the shaft. The performance and emissions were recorded by speed variations according to the SAE-J1349 standard for a speed range between 1300 and 3700 rpm. DYNO-MAX 2010 software was used for obtaining the output parameters.

The fuels used for experimentation were pure gasoline with an octane rating of 92 and gasoline blended with 10% by volume acetone (A10). Gasoline was arranged from Pakistan State Oil (PSO). In the current study, the 10% by volume of acetone was blended with 90% by volume of gasoline. Both fractions were mixed in the liquid phase. The ultrasonic bath was applied to evaluate the standardized fuel blend of acetone and gasoline for 30 min in order to ensure homogeneity. Phase stability was further assessed by two approaches: the visual method and the thermogravimetric analysis (TGA) technique. The visual technique relies on the phase observation of the prepared blend (A10). The tested mixture (single liquid phase) is kept in a long glass tube under ambient conditions for observation of phase stability. In the second approach, the vaporization behavior of the established fuels was assessed by employing the TGA. The temperature was steadily increased to permit the complete vaporization of the fuel components. The lowest temperature for operationability of acetone-blended gasoline fuel was found well below the standard temperature. The physicochemical attributes of the mentioned fuels are summarized in Table 2.

**Table 2:** Properties of Test Fuels

fuel property	acetone	A10	gasoline
molecular formula	C_3_H_6_O		C_8_H_15_
oxygen amount (% v/v)	27.6	2.76	0
research octane number	110	100	92
stoichiometric AF ratio	9.54	14.2	14.7
density (kg/m^3^)	0.791	0.751	0.745
heating value (MJ/kg)	29.6	41.2	42.7

The blended fuel was prepared by adding 10% per volume of acetone to 90% per volume of gasoline with the help of a cylindrical flask. The proper homogeneity of the fuel mixture had been ensured before experimentation was carried out. A calibrated measuring cylinder with 1% resolution was incorporated for supplying acetone to the carburetor. A probe of the gas analyzer (EMS-5002) was inducted into the exhaust pipe for a complete 60 s to ensure steady-state emission recording. The lubricant oil deterioration was also ascertained, along with performance and emission assessment for both fuels. The specific grade (SAE 20W-40) lubricant oil (properties as shown in Table 3) was used in the engine, as acclaimed by the manufacturer.

**Table 3:** Properties of Lubricating Oil

parameter	flash point °C	kinematic viscosity (cSt)	total base number (mg·KOH/g)	zinc (ppm)	phosphorus (ppm)
standards	ASTM D-92	ASTM D-445	ASTM-D2896	ASTM D-6595	ASTM D-6595
	159	17.5	8.9	902.6	851.89

The lubricating oil deterioration for the test fuels was quantified by operating the engine at 2800 rpm for 120 straight hours. After the designated time, the oil was hauled out from the oil sump and was examined according to the American Society for testing and materials (ASTM) standards, as mentioned in Table 3.

The uncertainty analysis can be used to determine the degree of measurement accuracy. Additionally, it provides the degree of inaccuracy in each experimental setup measurement. The quantifiable parameter range, accuracy, and uncertainty in the recorded readings are mentioned in Table 4. The total uncertainty of the experimental setup (*E*_exp_) can be ascertained through eq 1.^18^
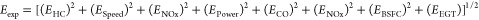
1
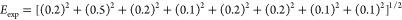

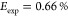


**Table 4:** Range and Accuracy for Measured Parameters

measured parameters	range	accuracy	uncertainty
power	0–50 kw	±0.05 kW	±0.1
NO_*x*_	0–5000 ppm	±1 ppm	±0.2
speed	0–8000 rpm	±5 rpm	±0.5
CO	0–18%	±0.01%	±0.2
EGT	0–1300 °C	±1 °C	±0.1
HC	0–5000 ppm	±1 ppm	±0.2
fuel consumption	0–100 mL	±0.1 mL	±0.1
CO_2_	0–18%	±0.1%	±0.2

## 3 Results

### 3.1 Performance Comparison of G and A10

#### 3.1.1 Brake Power

**Figure 2:**
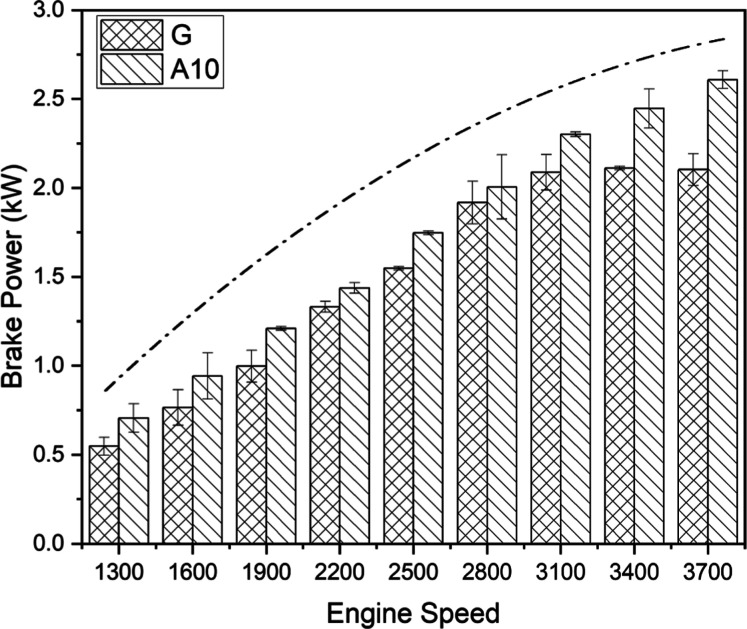
BP comparison for gasoline (G) and A10.

Pure gasoline and A10 (gasoline blended with 10% acetone) were assessed for comparative power production. The analysis unveils significantly better power generation with blended fuel than pure gasoline at all test speeds, as shown in Figure 2. BP possesses direct relation with torque and engine speed. Gasoline emerged 11.74% less efficient than A10 in terms of BP when employed in the engine. The boosted engine performance with the blended fuels could be attributed to a lean mixture of acetone and gasoline, increased fuel extraction efficiency of acetone due to the presence of oxygen, acetone’s high octane number, and the decreased tendency of knocking.^21,37^ The fuel droplet of acetone limits the evaporation and supports the improved combustion of the fuel blend, which contributes to enhanced power at the output shaft.^25^ As anticipated, acetone fuel showed the maximum BP at a speed of 3700 rpm. The growing dotted curve in Figure 2 signifies the engine’s general trend of BP variation when functioning at varying incremental speeds. The concave-up shape of the curve with a bow-like end explicates inherent power losses associated with higher operational speeds due to friction.^29^ It can be observed from Figure 2 that the BP curve became flat at higher engine speeds, but the curve for the acetone blend kept on increasing. This behavior might be due to better combustion for acetone-blended fuel. For gasoline, the friction rate relative to power generation might be higher at higher engine speeds, which is mainly responsible for the flatness of the BP curve. Intuitively, it might be apparent to increase the percentage of acetone in gasoline for further enhanced BP requirement; however, there is a limit of acetone addition to gasoline for efficient results. The higher BP in case of acetone-blended fuel matches with the finding of Alahmer.^22^

#### 3.1.2 EGT

**Figure 3:**
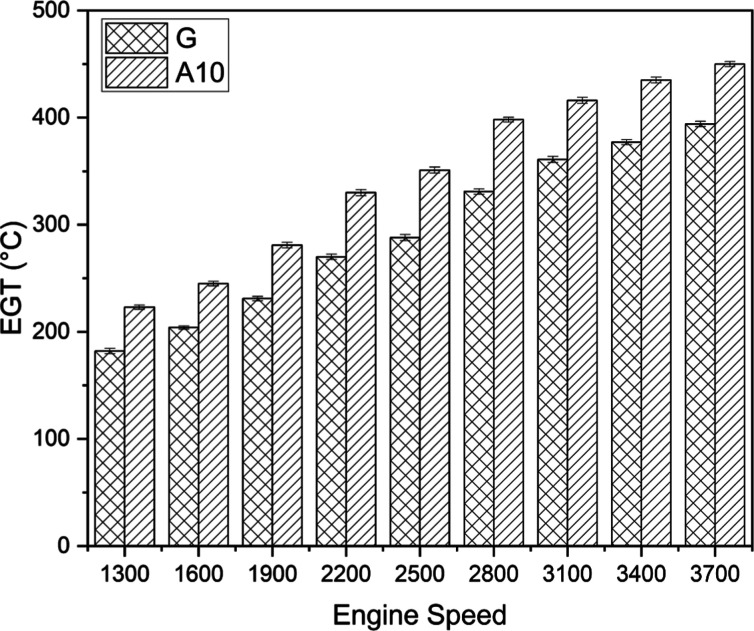
EGT comparison for gasoline (G) and A10.

EGT is the main indicator of the complete burning of fuel, and it exhibited direct relation with the appropriateness of fuel combustion. Figure 3 demonstrates the variation of EGT for two distinct fuels (gasoline and A10). It can be noticed that, generally, EGT kept on increasing with the rise in engine speed. The highest EGT of 394 and 450 °C was obtained for gasoline (G) and A10, respectively, at 3700 rpm. This engine behavior can be reasoned to more fuel consumption at higher speeds to fulfill higher power requirements. On average, the EGT of acetone-blended fuel (A10) was approximately 18.61% higher than that of gasoline. EGT assists in interpreting the evolution of exhaust emissions and understanding the combustion quality.^38^ When acetone-blended fuel is injected into the engine, a higher EGT implies efficient fuel burning inside the cylinder. The literature is contradictory when it comes to the EGT trend for acetone-blend fuels. It could go up or down depending on the amount of oxygen in the acetone and its latent heat of vaporization.^39^ Acetone possesses higher latent heat than gasoline, which is mainly responsible for higher VE and combustion rate, which ultimately results in higher EGT for acetone-blended fuels.^40^ Additionally, oxygenated acetone–gasoline fuel blends improve fuel combustion, combustion efficiency, and mixture strength, which produce more EGT.^19^

#### 3.1.3 Brake-Specific Fuel Consumption

**Figure 4:**
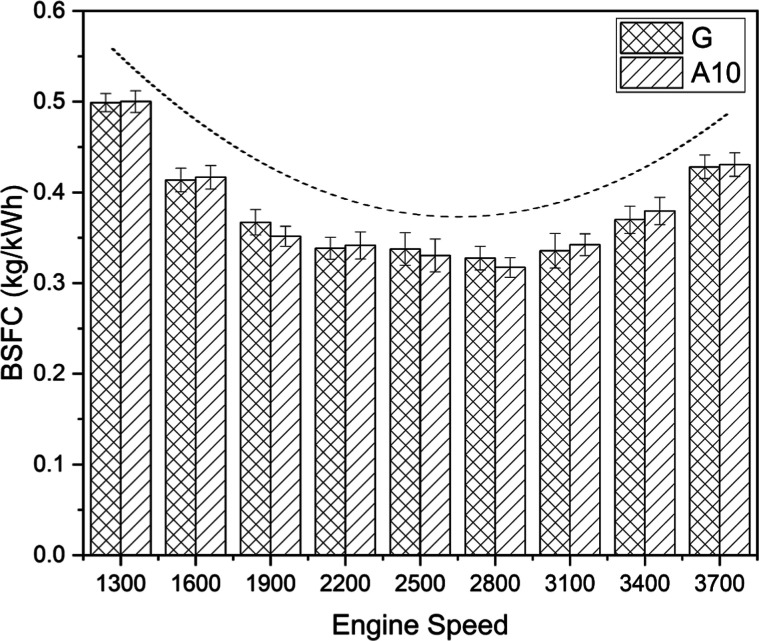
BSFC comparison for gasoline (G) and A10.

The variations in the BSFC of tested fuels with engine speed are noticeable in Figure 4. The fuel consumption pattern of an engine subjected to varied incremental speed could be rationally assessed by the overhead dotted curve. As the engine speed increases, BSFC first decreases before rising. More fuel was injected initially to get the engine running in order to counteract the effects of inertia. The heat loss from the engine’s cylinder walls was higher at lower engine speeds, which led to increased fuel consumption to make up for such losses. The BSFC progressively increased as engine speed increased, and then it started to rise again between 2500 and 2800 rpm. The combustion is close to stoichiometric when the BSFC for a given speed range is lowest. In order to satisfy the increased power need, the BSFC was enhanced for greater engine speeds. The abrupt lift in the curve at the culmination of experimental runs could be discerned by built-up frictional resistance at high speeds.^41^ On the comparative scale of fuel economy, acetone-blended fuel emerged meaningfully favorable owing to an average 6.72% reduced BSFC. At the test speed of 2800 rpm, gasoline was declared 8.38% less efficient than its competitor (A10). Both fuels showed the highest BSFC at the lowest speed, that is, 1300 rpm. The less fuel consumption of A10 in comparison with gasoline at 2800 rpm could be accredited to the following reasons: (a) lower energy density of blended fuel and (b) higher latent heat of vaporization of acetone.^21,42^

#### 3.1.4 Brake Thermal Efficiency

**Figure 5:**
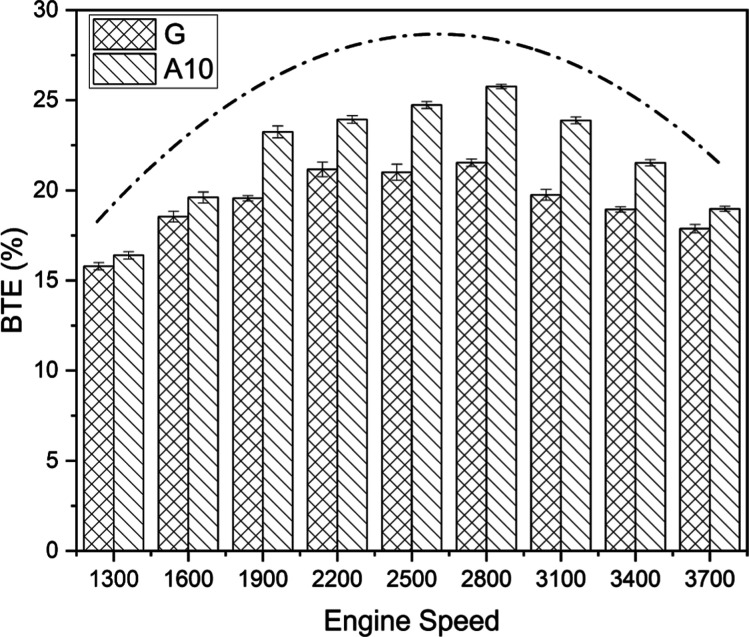
BTE comparison for gasoline (G) and A10.

The performance of an engine with two different fuels (G and A10) is graded by the engine BTE in Figure 5. The vital assessment parameter, BTE, rendered acetone-blended fuel apposite on account of a 12.05% higher thermal efficiency when juxtaposed with pure gasoline. The fuels under consideration showed the peak value of BTE at the engine’s speed of 2800 rpm, with A10 being 16.38% more promising than its contender (100% gasoline). The boost in engine thermal efficiencies with acetone-blended fuel is due to higher latent heat and lower fuel evaporation of acetone than those of gasoline.^23,43^ The rising–falling dotted–dashed curve in the figure under discussion is an image-based depiction of the variation of thermal efficiencies of an engine at various speeds. The decline after the zenith value gives an essential insight into engine operation at high speeds. Generally, increased revolutions of the engine shaft are accompanied by decreased completion time of combustion. Thus, the engine demands more fuel to produce the desired output, reducing BTE, as indicated by a downward-bent portion of the curve.^44^ Additionally, it is evident that there was a certain speed range where fuel transformation efficiency to useful work was at its peak and fuel consumption was at a minimum constant. After reaching its maximum range, BTE began to decrease as a result of increased losses and a need for greater power at higher engine speeds. The higher BTE in case of acetone-blended fuel (A10) is caused by a higher power-to-fuel consumption ratio.

### 3.2 Emission Comparison of G and A10

In this section, the environmental hazards caused by CO, CO_2_, HC, and NO_*x*_ emissions from fuels under test have been described in detail.

#### 3.2.1 CO Emission

**Figure 6:**
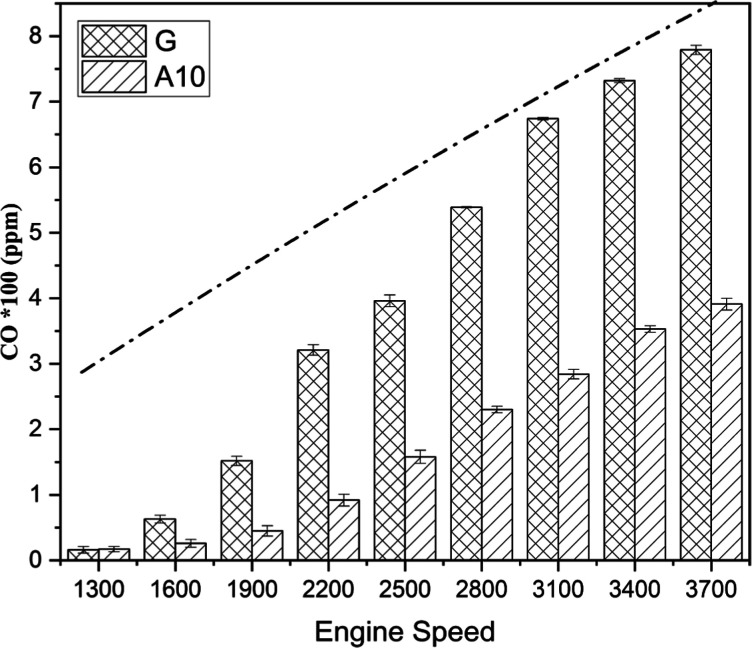
CO emission comparison for gasoline (G) and A10 at different engine speeds.

Carbon monoxide (CO) is a harmful pollutant and is undesirable for a clean environment. Figure 6 shows the relationship between engine speed and the overall rising trend in CO emissions. It increases by the engine components moving with more inertia and the insufficient mixing of the molecules of fuel and air. Additionally, a larger percentage of fuel particles being expelled after partial reaction with oxygen is to blame for an increase in CO emissions. Once again, A10 outperformed its competing fuel (gasoline) as acetone in gasoline reduced exhaust emission of CO by 56.54%. At a test speed of 3700 rpm, G produced 7.79 ppm of CO, while A10 marked the value of 3.91 ppm on the measuring scale. The peak value of tailpipe emission was recorded at the highest speed, that is, 3700 rpm, indicating the substantial influence of engine speeds on pollutant emissions.^45,46^

The reduced emission of acetone-blended fuel than neat gasoline is due to oxygen content, complete combustion, and lower carbon content in acetone.^47−50^ The dotted curve shows the trend of CO emission of the SI engine. The curve solely rises due to incomplete fuel combustion at high engine speeds.

The obtained results are consistent with previous studies. Elfasakhany^51^ conducted an experiment on a 147.1cc SI engine by employing four test fuels. He obtained 45, 28, and 21% lower CO emissions for ACE10, ACE7, and ACE3, respectively, compared with gasoline at 3000 rpm. In another set of experiments,^52^ the author obtained about 46.7, 44.5, and 40% lower CO emissions for ACE10, ACE7, and ACE3, respectively, compared with gasoline at fixed 2600 rpm. Table 5 indicates that the mean value of CO emission for gasoline (G) is relatively higher than that for A10.

**Table 5:** Average CO and CO_2_ Contents for the 95% Confidence Interval

	carbon monoxide [CO (ppm)]			carbon dioxide [CO_2_ (%)]		
fuel	mean ± Std. dev	skewness	mean ± 95% CI	mean ± Std. dev	skewness	mean ± 95% CI
G	408 ± 2.91	–0.08	408 ± 2.24	7.47 ± 1.94	–0.34	7.47 ± 1.5
A10	177 ± 1.43	0.32	177 ± 1.11	4.96 ± 1.16	–0.37	4.96 ± 0.89

**Figure 7:**
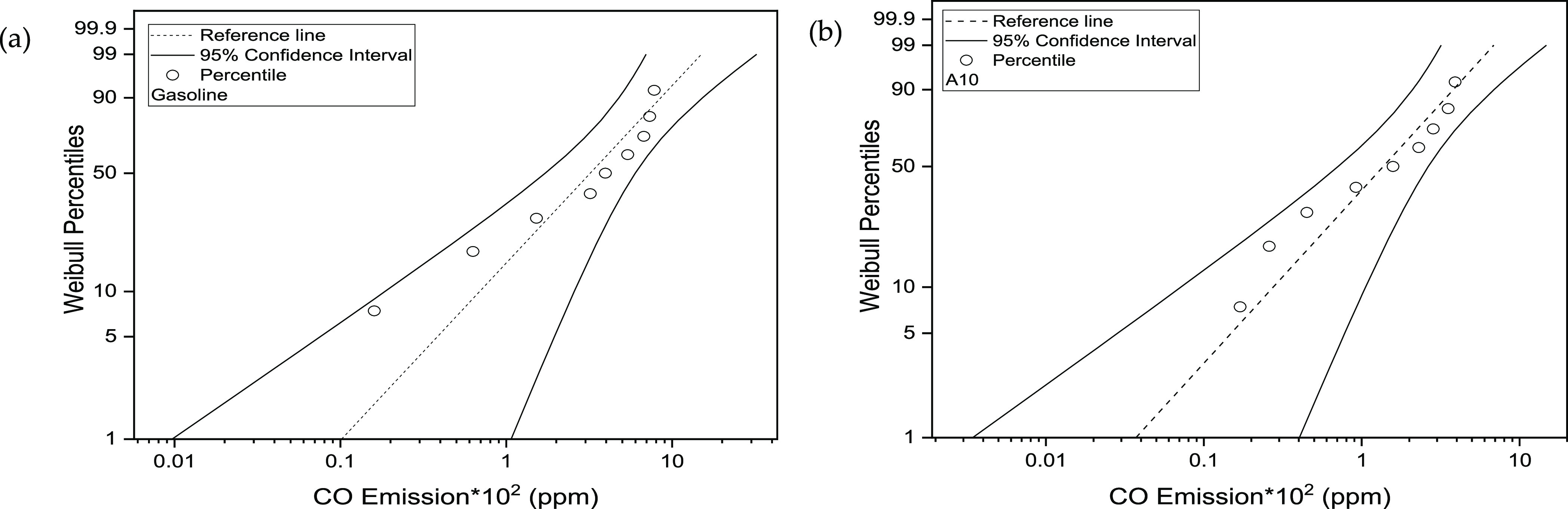
(a) CO emission Weibull probability against 95% CI for gasoline; (b) CO emission Weibull probability against 95% CI for A10.

The CO emission data in the case of gasoline, when fitted for the 95% confidence interval (CI), the 50th percentile, varies from 1.71 to 5.98 ppm with respect to the optimal range of minus 33.77% to plus 56.81%, while the CO emission data in the case of A10, when fitted for 95% CI, the 50th percentile varies from 0.708 to 2.64 ppm with respect to the optimal range of minus 40.15% to plus 55.18%. The percentage of data lying within the designated CI verifies the authenticity of statistically plotted data. The fitted data was bounded between the designated limits against selected CIs. The fitted data did not depict any heavy tail around the distribution, again showing the goodness of data fitting. It is not mandatory to exhibit symmetric nature of mean data points. They can also be unsymmetric in nature. It can be noticed from Figure 7 that CO emission for gasoline is skewed negatively, which depicts a longer tail on the left portion of the distribution. For A10, CO emission is skewed positively, indicating stretching of the tail along the right portion of the distribution. The skewness indicates the unsymmetrical nature of the distribution.

#### 3.2.2 CO_2_ Emission

**Figure 8:**
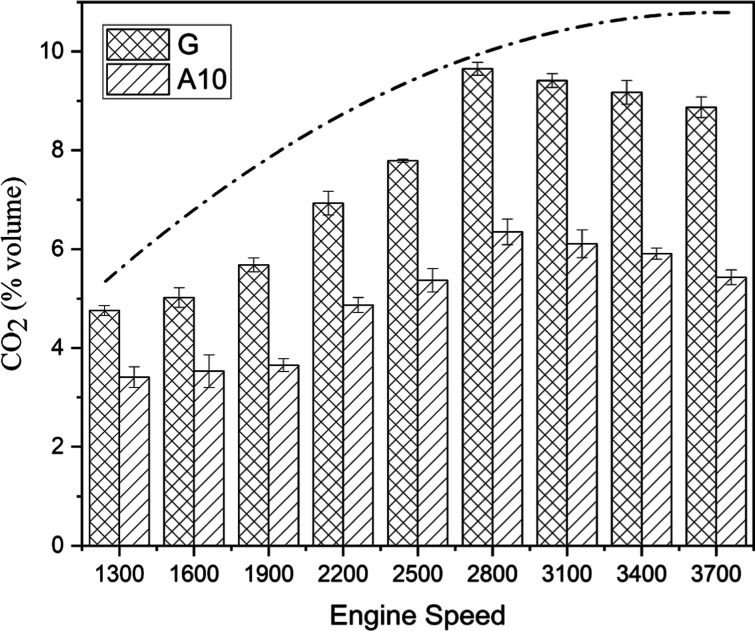
CO_2_ emission comparison for gasoline (G) and A10 at different engine speeds.

The variation in CO_2_ as greenhouse gas emission^39^ from an engine operating on two fuels separately is shown in Figure 8. The growing–falling dotted curve epitomizes the tailpipe exhaust pattern of greenhouse pollutants in Figure 8. The movement along the abscissa was found to be directly correlated with the movement along the ordinate up to the maximum speed of 2800 rpm, after which the curve incurred an abrupt descent.

The acetone addition to gasoline has noticeable efficacy due to an average of 33.67% lesser contribution to percentage volume emission than pure gasoline. Among the test runs, the most considerable variation in emission occurred at 2800 rpm, with gasoline and A10 sharing 9.65 and 5.93% of the total volume of gas emitted. Complete combustion results in the production of CO_2_, which is directly related to the BTE. The CO_2_ emission would be higher for fuel that burns more efficiently. If not, fuel would burn less efficiently, lowering CO_2_, and increased CO emissions would result. By converting CO to CO_2_, the existence of oxygen subsequently encourages lean burning and enhances combustion. CO_2_ emission is contingent on the carbon–hydrogen ratio of the fuel or oxygen content. The CO_2_ formation could be apprehended by considering the carbon atoms in the fuel’s molecules. Acetone has three carbon atoms while gasoline has eight carbon atoms, and therefore, the reduction in the carbonaceous emission is practically obvious for A10 as acetone possesses higher oxygen and lower carbon content in reference to gasoline.^23^ Therefore, a carbon-to-oxygen ratio decrease is mainly responsible for lower CO_2_ emission.^53^ The obtained results are also in accordance with previous studies. Elfasakhany^51^ obtained 34, 41, and 45% lower CO_2_ emissions for ACE10, ACE7, and ACE3, respectively, compared with gasoline at 3000 rpm. In another set of experiments, he^52^ obtained about 35.5, 34, and 29.5% lower CO_2_ emissions for ACE10, ACE7, and ACE3, respectively, compared with gasoline at fixed 2600 rpm. The mean value of CO_2_ emission for A10 is relatively lower than that for G (see Table 5).

**Figure 9:**
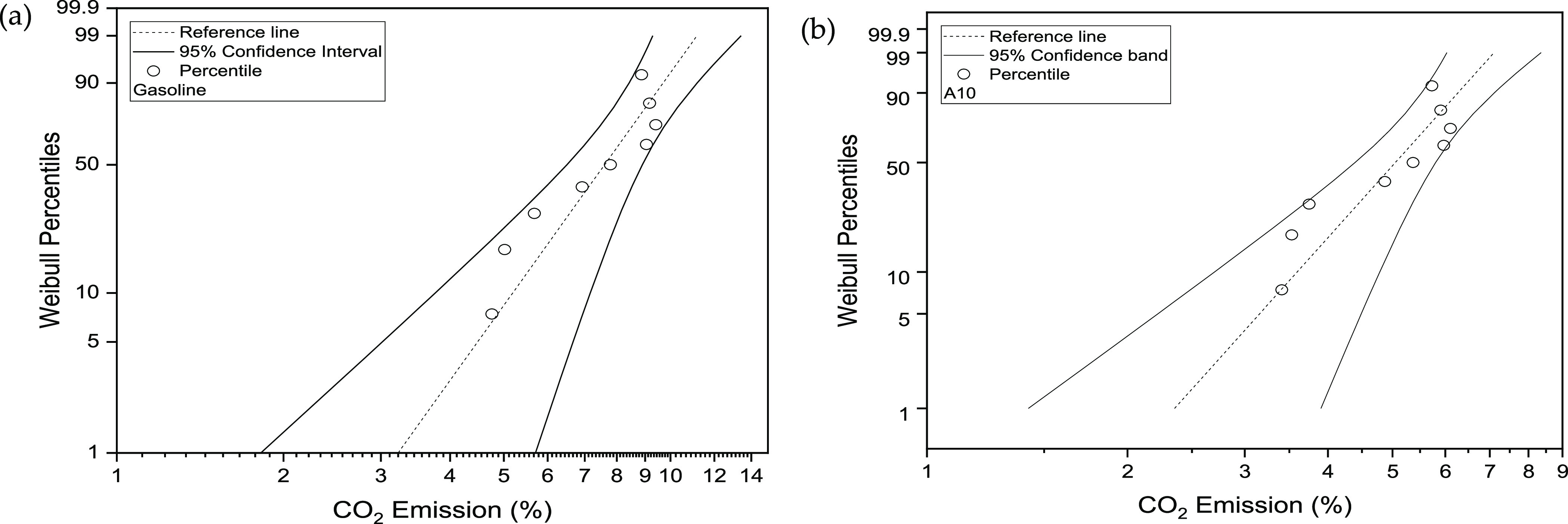
(a) CO_2_ emission Weibull probability against 95% CI for gasoline; (b) CO_2_ emission Weibull probability against 95% CI for A10.

The 50th percentile of CO_2_ emission under 95% CI for G fluctuates from 6.49 to 8.89%, concerning the optimal range of negative 12.37% to positive 16.68%. In comparison, the 50th percentile (CO_2_ emission) in the case of A10 against 95% CI fluctuates from 4.39 to 5.80% with respect to the optimal range of negative 7.41% to positive 18.24%. The validity of statistically plotted data can be confirmed by considering the amount of data falling within the selected 95% CI. The fitted data was bounded between the designated limits against selected CIs. The fitted data did not depict any heavy tail around the distribution, which again shows the goodness of data fitting. From Figure 9, the CO_2_ emission for both G and A10 is skewed negatively, indicating a longer tail toward the left portion of the distribution. The skewness indicates the unsymmetrical nature of the distribution.

#### 3.2.3 HC Emissions

**Figure 10:**
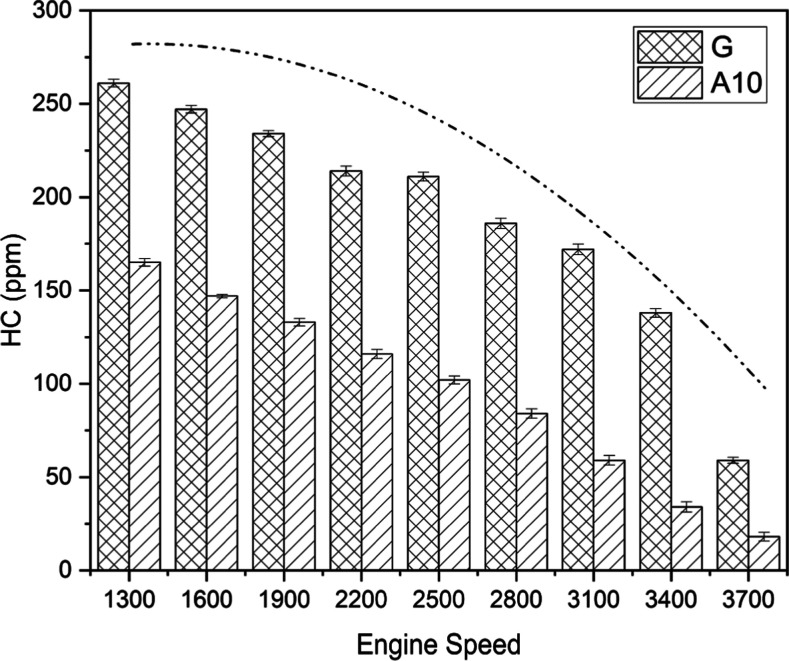
HC emission comparison for gasoline (G) and A10 at different engine speeds.

Hydrocarbon (HC) emissions are considered to be one of the significant environmental burden indicators.^41^ HC emissions of test fuels are comprehensively shown in Figure 10.

Both fuels showed an overall decreasing trend of HC emissions with continuous increases in engine speed. The higher combustion temperature inside the cylinder allowed for quicker fuel combustion with less flame quenching to the cylinder walls and adsorption or desorption in the oil layer, which can be the cause for the general trend of HC emission decreasing with engine speed. The acetone-blended gasoline emerged as more friendly to the environment due to the average emission magnitude being half lower than that of neat gasoline. The dashed–dotted curve graphically shows the emission patterns of G and A10. It could be sanely deduced that the hydrocarbon emission decreases with augmented engine speed for both fuels. Moreover, the worst HC emissions for test fuels were found to be at minimum speed, that is, 1300 rpm. As clear from the name, the unburnt hydrocarbons are produced due to incomplete combustion inside the engine chamber and disappear with better combustion at higher speeds.^29^ The blended fuel, acetone, has oxygen content present, facilitating improved combustion and could be reasonably attributed to the better performance in terms of HC emission comparable to unblended gasoline.^24,54^ The fundamental cause of the lower production of HC emissions is thought to be hydrocarbon fuel oxidation in the postflame as a result of blending with oxygenated fuel.^55^ Because oxygen reacts with hydrogen to make H_2_O and with carbon to produce CO_2_, the oxygen proportion in methanol promotes clean combustion.^38^ Since there is less reactivity between hydrogen and carbon, there are fewer HC emissions. This decline in HC emission coincides with the previous research.^56^Table 6 depicts a higher HC emission mean value for G than for A10.

**Table 6:** Average HC and NO_*x*_ Contents for the 95% Confidence Interval

	hydrocarbon [HC (ppm)]			nitrogen oxides [NO_*x*_ (%)]		
fuel	mean ± Std. dev	skewness	mean ±95% CI	mean ± Std. dev	skewness	mean ±95% CI
G	191.33 ± 62.68	–1.22	191.33 ± 48.18	528.56 ± 272.47	–0.11	528.56 ± 209.43
A10	95.33 ± 16.9	0.32	95.33 ± 39.01	876.33 ± 436.52	0.17	876.33 ± 335.55

**Figure 11:**
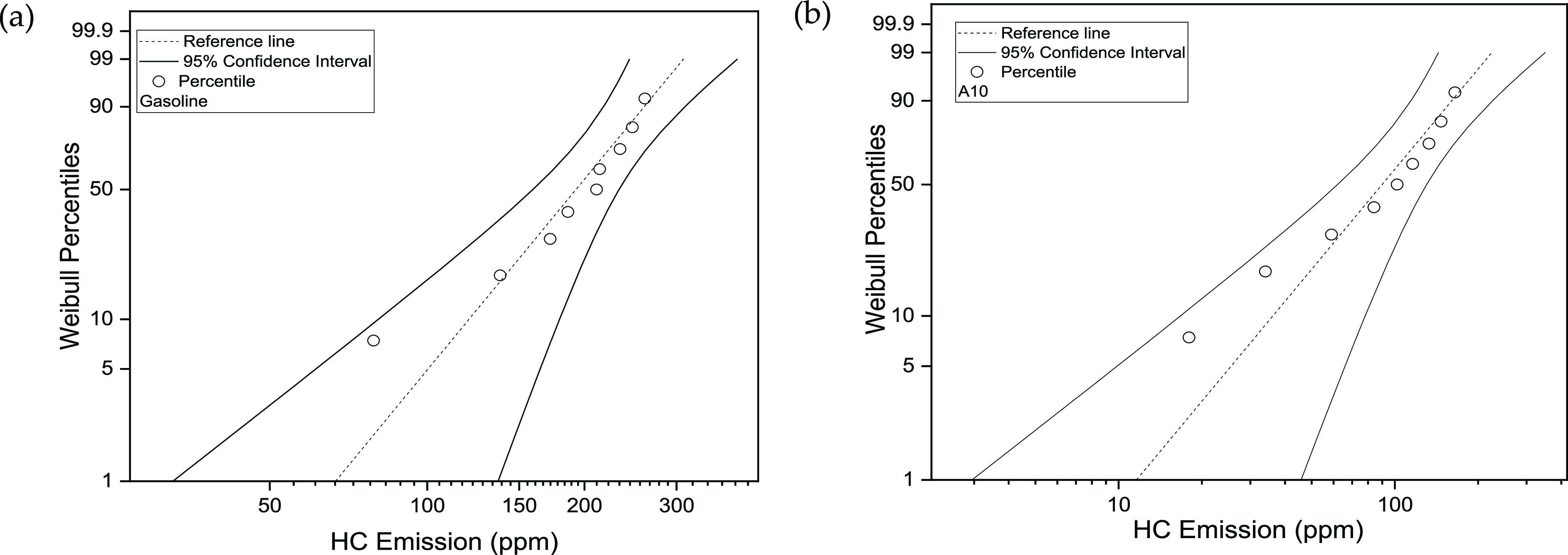
(a) HC emission Weibull probability against 95% CI for gasoline; (b) HC emission Weibull probability against 95% CI for A10.

The 50th percentile of HC emission under 95% CI for G fluctuates from 159.31 to 232.89 ppm, concerning the optimal range of negative 9.39% to positive 24.49%. In comparison, the 50^th^ percentile (HC emission) in the case of A10 against 95% CI fluctuates from 61.99 to 130.28 ppm, concerning the optimal range of negative 21.70% to positive 39.22%. The validity of statistically plotted data can be confirmed by considering the amount of data falling within the selected 95% CI. The fitted data was bounded between the designated limits against selected CIs. The fitted data did not depict any heavy tail around the distribution, again showing the goodness of data fitting. It is evident from Figure 11a,b that HC emission for gasoline is negatively skewed, which depicts longer distribution on the left side of the tail. However, for A10, HC emission is positively skewed, which means the tail on the right side of the distribution is longer. The skewness indicates the unsymmetrical nature of the distribution.

#### 3.2.4 NO_*x*_ Emissions

**Figure 12:**
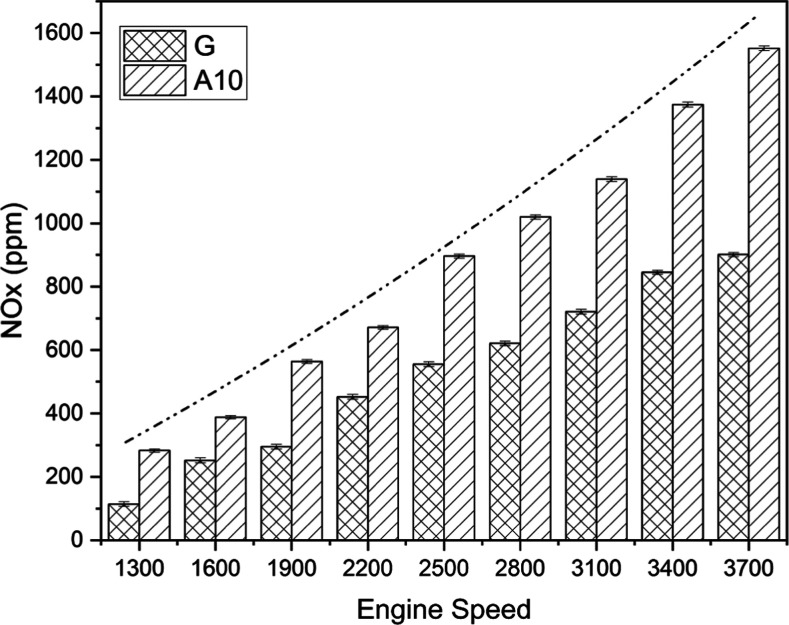
NO_*x*_ emission comparison for gasoline (G) and A10 at different engine speeds.

One of the essential emissions associated with fuel combustion inside an engine is nitrogen oxide. The impact of speed on NO_*x*_ emission is graphically portrayed in Figure 12.

Unlike CO, CO_2_, and HC emissions, acetone-blended gasoline emerged unfavorable due to 39.66% higher NO_*x*_ emissions than pure gasoline. Moreover, the lowest and maximum test fuel emissions were observable at the extremes of the speed range, that is, 1300 and 3700 rpm, respectively. Nitrogen oxide formation is directly associated with the temperature inside the cylinder.^57^ The oxygen content of acetone aids quick and improved combustion and consequently increases the cylinder temperature, which ultimately augments NO_*x*_ formation. Any engine operating at high speeds will result in an obvious increase in cylinder temperature, which is shown by the rising dashed–dotted curve.^58^ Moreover, the observed NO_*x*_ increase may also be associated with a decrease in CO_2_ emissions at high speeds.^45−47^Table 6 depicts that the mean value of NO_*x*_ emission for G is relatively lower than that of A10. The breakdown of diatomic nitrogen molecules into highly reactive monoatomic nitrogen can be used to explain the greater NO_*x*_ emission. NO_*x*_ emissions are created when monoatomic nitrogen and oxygen in the mixture of air and fuel react. EGT aids in interpreting the development of exhaust emissions and understanding the quality of combustion.^38^ The main justification for additional fuel injection into the cylinder is the reduced heating value of gasoline combined with acetone. As a result of burning more oxygenated fuel, a greater EGT was produced. The reaction between oxygen and monoatomic nitrogen is catalyzed by the increased temperature within the engine cylinder, depicted by higher EGT, leading to higher NO_*x*_ production for acetone-blended fuel.

**Figure 13:**
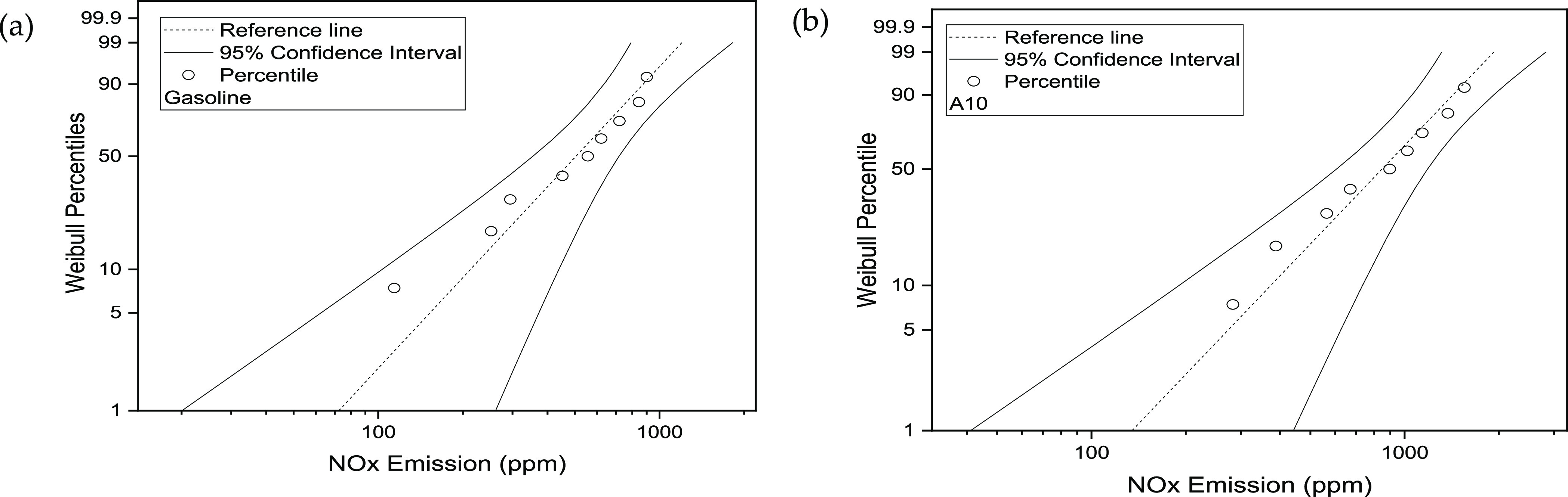
(a) NO_*x*_ emission Weibull probability against 95% CI for gasoline; (b) NO_*x*_ emission Weibull probability against 95% CI for A10.

The 50th percentile of NO_*x*_ emission under 95% CI for G fluctuates from 354.33 to 717.38 ppm, concerning the optimal range of negative 22.49% to positive 36.27%, while the 50^th^ percentile (NO_*x*_ emission) in the case of A10 against 95% CI fluctuates from 605.54 to 1182.91 ppm with respect to the optimal range of negative 24.25% to positive 32.41%. The validity of statistically plotted data can be confirmed by considering the amount of data falling within the selected 95% CI. The fitted data was bounded between the designated limits against selected CIs. The fitted data did not depict any heavy tail around the distribution, which again shows the goodness of data fitting. It is evident from Figure 13 that NO_*x*_ emission for gasoline is negatively skewed, which means the tail on the left side of the distribution is longer. For A10, NO_*x*_ emission is positively skewed, which indicates a longer tail on the right side of the distribution. The skewness indicates the unsymmetrical nature of the distribution.

### 3.3 Lubricating Oil Deterioration

#### 3.3.1 Alteration of Physical and Chemical Properties

**Figure 14:**
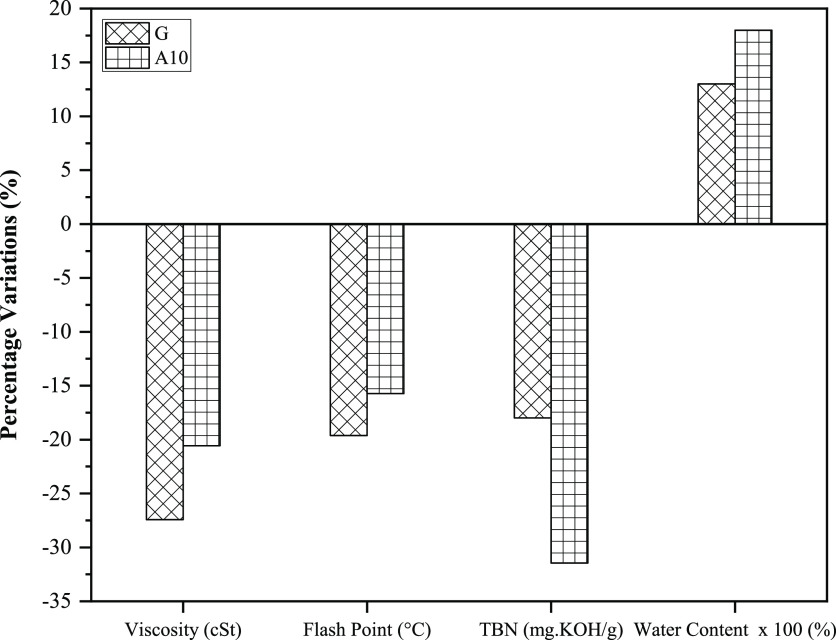
Comparison of lubricating oil properties for G and A10.

Engine lubricating oil is central to an engine’s smooth and efficient working. It decreases the friction among moving/reciprocating parts and thus influences the engine performance.^59^ In this section, the comparative effect of gasoline and A10 on lube oil’s physical and chemical degradation has been thoroughly investigated. The variations in the properties, kinematic viscosity (KV) at 100 °C, FP, TBN, and water content have been evaluated by comparing them to the properties of fresh oil. Figure 14 comprehensively depicts the influence of gasoline and A10 on lubricating oil properties after 120 straight hours of engine operation.

The datum or zero reference line indicates the properties of fresh oil. Moreover, for G and A10, the negative and positive y-regions designate the decrease and increase from the reference value. KV is a vital attribute of lubricating oil regarding friction control, fuel efficiency, and emissions. Any slight variations in it would have considerable repercussions, often associated with the breakdown of large molecules and fuel dilution.^58^

Because of the unavailability of lubricant oil layers between reciprocating parts, it would be difficult to sustain the frictional load, and consequently, more friction eventually leads to wear.^31^Figure 14 shows that after engine operation for the designated time, the KV decreased by 27.43 and 20.57% for G and A10. However, the rate of decrease for A10 was 25% lower compared to G, which renders A10 less detrimental to lube oil. The KV of lubricant oil was ascertained through the ASTM D445 standard. The lubricant oil should possess an optimum KV value. If the KV would be higher, then engine power will be consumed to pump lubricant oil inside an engine, resulting in a decline in net power. However, if the kinematic viscosity would be lower, then the lubricant oil is unable to cover the space between mating parts, and ultimately the friction will increase.^60^ The lower decline in KV for acetone-blended gasoline could be attributed to oxide formation and mixing of sludges.^29^

The FP of lubricating oil served to be the threshold temperature at which the vapors are ignited when provided with the spark. The FP of lubricant oil for distinct test fuels was ascertained by following ASTM D92. As FP regulates the fire safety of oil applications, it affects the maximum operating limit of lubricating oil. The lower FP denotes a potential risk of lubricating oil during system operation, which could lead to a malfunction.^10^ The percentage variations for gasoline and A10 advocate the decline of 19.63 and 15.73% in FP equated to fresh oil run, respectively. Moreover, the decline of pure gasoline was 19.88% higher than that of A10. Thus, once again, the fewer variations in FP vouched for A10 as more potential fuel for guarding the earlier oil deterioration. The fuel dilution concept could ascertain the decline in flash points.^61^

Similarly, the TBN variations of fuels under study are also shown in Figure 14. The alkaline derivatives that exist in lubricating oil may govern its serviceability and are indicated by the TBN value of the oil. A lower TBN number indicates poor performance and more corrosion. A higher TBN, however, suggests improved antioxidation capabilities.^62^ The alkaline nature of a lubricant is gauged by TBN, and it is desirable to be high for efficient performance and corrosion prevention.^50^ The ASTM D-2896 standard was followed to ascertain the TBN of lubricant oil. The percentage variations in TBN for G and A10 were 17.98 and 31.46%, respectively, compared to nondeteriorated oil. Unlike KV and FP, the A10 proved detrimental to lube oil owing to a higher decline in TBN compared to G. The water content variations of test fuels are shown on an exaggerated scale (Figure 14). The ASTM D-95 standard was followed to determine moisture (water) content in lubricant oil. Moisture contaminates the lubricant oil when suspended in it, causing chemical and physical issues among engine parts and operationability. The factors which are responsible for moisture in the lubricant oil are rusting of engine parts, disruption in lubricant oil film, oxidation, embrittlement of hydrogen, and water etching.^63^ The water content of lubricant oil indicates contamination from external sources. In a percentage variation pattern dissimilar to other properties, the water % increased for oil run on both fuels. However, the increase was observed to be higher in the case of acetone-blended gasoline. Thus, the comparative alteration of physical and chemical properties of lube oil with two fuels declares that A10 might be undesirable in certain aspects of engine oil damage.

#### 3.3.2 Contamination by Suspended Particles

**Figure 15:**
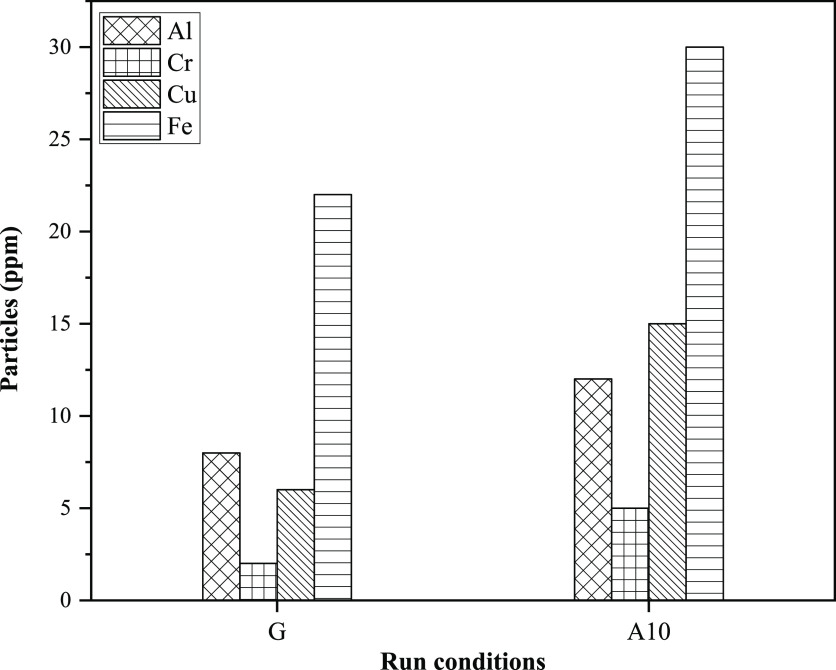
Comparison of wear debris for G and A10.

The oxidation process itself and the products formed are unfavorable for lubricating oil and could significantly deteriorate it. The existence of foreign particles turned into excessive oxidation, frequently uncontrollable, and must be taken into account.^64^Figure 15 shows the lubricating oil deterioration with gasoline and the blend of gasoline with 10% acetone in terms of the occurrence of suspended particles, that is, chromium (Cr), copper (Cu), aluminum (Al), and iron (Fe).

The mechanical parts inside automobiles are mostly composed of iron-based alloys and the wearing of bearings, crankshafts, piston rings, cylinder valves, and so forth, mainly responsible for the presence of iron (Fe) in lubricant oil. Aluminum (Al) is mainly used in manufacturing journal and piston jackets because of its higher heat transfer rate and lower density. The lubricant oil with aluminum particles indicates wear in the piston. Copper alloys are used in manufacturing intermediate layers of engine bearings, and the existence of copper in the lubricant oil indicates wear in these layers. The potential sources of chromium (Cr) in lubricant oil as wear debris are piston rings, cylinder liners, and crankshafts.^65^ The ASTM D-6595 standard was followed to determine the wear debris in lubricant oil through a spectrophotometer manufactured by SpectrOil.

The straightaway visual comparison unveils that blended fuel caused a considerably higher occurrence of all suspended particles compared to pure fuel. Among all particles, the concentration of iron was ascertained to be maximum for gasoline (22 ppm) and A10 (30 ppm), followed by copper, chromium, and aluminum in descending order. The comparison of increment in suspended particles with fresh oil shows that for gasoline, Al, Cr, Cu, and Fe increased by 8, 2, 6, and 22%, respectively. Moreover, A10 behaved significantly poorer with a 12, 5, 15, and 30% increase in Al, Cr, Cu, and Fe, respectively, which could be apprehended by excessive fuel compared to blended gasoline.^17^

#### 3.3.3 Wearing of Additives

**Figure 16:**
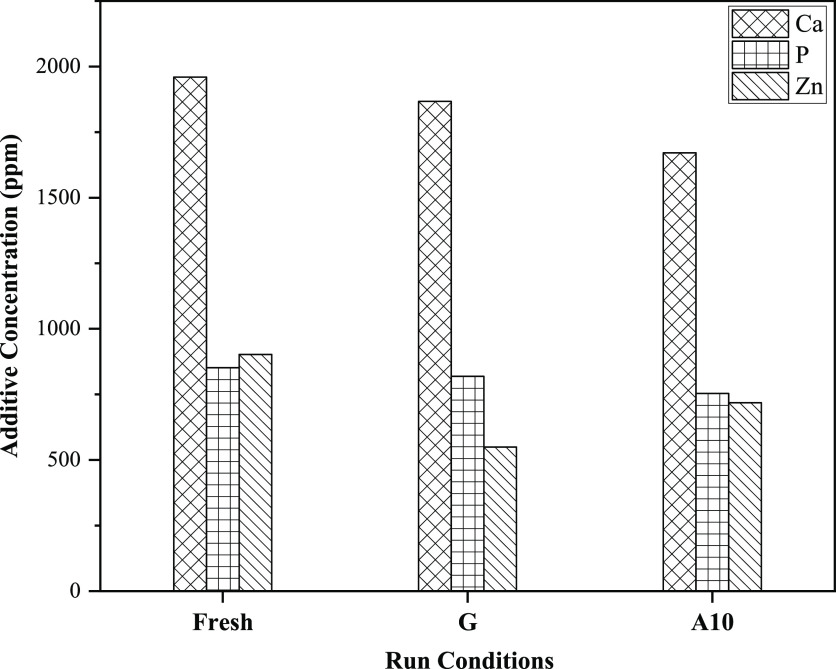
Additive depletion rate of G and A10 compared to fresh oil.

Additives are the heart and soul of the composition of lubricants. Each additive is designated to perform a specific function, and the absence or decline of any additive from a specific value would be an obvious deterioration. During the operation of an engine, the major deterioration comes from wearing useful additives.^66,67^Figure 16 shows the comparative evaluation of calcium, phosphorus, and zinc additive for the fresh oil and for lubricant oil used on gasoline and A10 running conditions.

The proportion of performance additives in lubricant oil can be found through additives depletion analysis. Zinc is a component of the antiwear particle in lubricating oils that provide a lower friction coating to protect the metal. In lubricating oil, calcium is a component of the detergent additives employed to neutralize combustion byproducts with an acidic character.^62^ The phosphorus in lubricant oil acts as an antiwear additive through the formation of a thin protective layer on metal parts. Once again, acetone addition to gasoline proved unfavorable because of the higher depletion of Ca and P than gasoline. However, zinc depletion has the reverse case. Compared to fresh lube oil for gasoline running conditions, Ca, P, and Zn decreased by 4.74, 3.86, and 39.15%, respectively, with Zn incorporating the most significant depletion. Similarly, for A10, the depletion rates were 14.75, 7.90, and 20.37% for Ca, P, and Zn, respectively.

Therefore, on the overall grounds, gasoline emerged to have a lower depletion rate of metal additives associated with blended fuel for 120 h of engine operation.

## 4 Conclusions

This work compares pure gasoline and 10% acetone-blended gasoline for performance, emissions, and lubricating oil deterioration. The outcomes are summarized as follows:

- Engine operating with A10 generates 11.74% higher BP than neat gasoline.
- Gasoline appears less efficient than A10, owing to a 6.74% higher BSFC and a 12.05% reduced BTE.
- A10 emerges as less damaging to the environment because of 56.54, 33.6 7, and 50% lower CO, CO_2,_ and HC emissions than its competitor.
- NO_*x*_ emissions of blended fuel are higher than that of neat fuel.
- KV and FP of lubricating oil decreased by 27.43 and 19.63% for gasoline and 20.57 and 15.73% for A10 compared to fresh oil. However, the TBN decline percentage concerning fresh oil is higher for A10 (31.46%) than for gasoline (17.98%).
- A10 is more detrimental to lubricating oil due to a 12, 5, 15, and 30% increase in Al, Cr, Cu, and Fe, respectively, compared to fresh oil.
- Ca, P, and Zn declined by 4.74, 3.86, and 39.15% for gasoline compared with fresh oil. For A10, there is a decline of 14.75, 7.90, and 20.37% in Ca, P, and Zn compared to fresh lubricant oil.

The detailed assessment of acetone as an alternative blended fuel in a SI engine proved valuable for optimized performance and reduced exhaust emissions. However, the impact of acetone addition proved unfavorable for lubricating oil operations and could impart early damage and life cycle reduction. Therefore, the possible damage due to waste lube spills should be potentially accounted for while considering acetone as an alternative fuel. In the future, the composition of lubricating oil should be chemically manipulated according to the combustion behavior of acetone for optimized outcomes. Additionally, it is necessary to develop such coatings for current engine metallurgy or develop new materials for the engine and its accessories which resist wear and tear when acetone-blended fuel is used. This will prevent wear particles from mixing with lubrication oil and slow down the rate of deterioration. Additionally, an engine’s life and performance will improve with less internal wear and tear.
